# The Impact of a Sparse Migration Topology on the Runtime of Island Models in Dynamic Optimization

**DOI:** 10.1007/s00453-017-0377-2

**Published:** 2017-09-20

**Authors:** Andrei Lissovoi, Carsten Witt

**Affiliations:** 1 Department of Computer Science, University of Sheffield, Sheffield, UK; 2 DTU Compute, Technical University of Denmark, Kongens Lyngby, Denmark

**Keywords:** Evolutionary algorithms, Island models, Dynamic problems, Populations, Runtime analysis

## Abstract

Island models denote a distributed system of evolutionary algorithms which operate independently, but occasionally share their solutions with each other along the so-called migration topology. We investigate the impact of the migration topology by introducing a simplified island model with behavior similar to $\lambda$ islands optimizing the so-called Maze fitness function (Kötzing and Molter in Proceedings of parallel problem solving from nature (PPSN XII), Springer, Berlin, pp 113–122, 2012). Previous work has shown that when a complete migration topology is used, migration must not occur too frequently, nor too soon before the optimum changes, to track the optimum of the Maze function. We show that using a sparse migration topology alleviates these restrictions. More specifically, we prove that there exist choices of model parameters for which using a unidirectional ring of logarithmic diameter as the migration topology allows the model to track the oscillating optimum through *n* Maze-like phases with high probability, while using any graph of diameter less than $c\ln n$ for some sufficiently small constant $c>0$ results in the island model losing track of the optimum with overwhelming probability. Experimentally, we show that very frequent migration on a ring topology is not an effective diversity mechanism, while a lower migration rate allows the ring topology to track the optimum for a wider range of oscillation patterns. When migration occurs only rarely, we prove that dense migration topologies of small diameter may be advantageous. Combined, our results show that the sparse migration topology is able to track the optimum through a wider range of oscillation patterns, and cope with a wider range of migration frequencies.

## Introduction

Optimization problems are often dynamic in nature, as the environment in which they have to be solved may change with the passing of time. Nature-inspired algorithms are based on approaches to solving optimization problems observed in nature, and we might therefore hope that they would also provide a reasonable solution to coping with dynamic changes in optimization problems. The performance of nature-inspired algorithms on dynamic problems has been considered in the literature [1, 19], including a number of runtime analyses of evolutionary algorithms on dynamic problems [3, 5, 8–10, 20].

In a dynamic optimization problem, the optimum is allowed to move in the search space over time, as conditions of the problem change. The goal of the optimization algorithm is then not only to locate the optimum once, as in the case of static optimization problems, but also be able to track the optimum as it moves, maintaining good solutions over time.

With the emergence of massively parallel computer architectures, parallel implementations of nature-inspired algorithms have become increasingly popular. A wide-spread approach called *island models* runs several instances of the same algorithm, the so-called *islands*, in parallel, with synchronization and exchange of information controlled by the length of the so-called *migration interval*. The topology of the network describing the information exchange is called *migration topology.* It is empirically well known [2, 21] that both the choice of migration interval and topology are crucial for the performance of the island model.

Despite the huge empirical knowledge, theoretical studies of the impact of the parameters of island models have only recently been published. Lässig and Sudholt [12] presents an example where the proper choice of the migration interval provably speeds up the runtime by an exponentially large factor. Mambrini and Sudholt [18] proposes an adaption scheme for the choice of the migration intervals and present a framework for theoretical runtime bounds. Lissovoi and Witt [17] is one of the few works showing the utility of island models on an in fact dynamic optimization problem from a theoretical perspective. The dynamic problem considered there is Maze, a pseudo-Boolean fitness function.

The Maze function, first introduced in [11], is an artificial fitness function defined over *n*-bit strings. It consists of $n+1$ long phases, over the course of which the optimum slowly shifts from the all-ones bit string to the all-zeros bit string, while oscillating between two specific solutions during each phase. In [11], it is shown that a simple (1 $+$ 1) EA is not able to track the oscillating optimum through all $n+1$ phases. Subsequent work [15, 17] has considered how various diversity mechanisms impact the ability of evolutionary algorithms to track the optimum of this function, observing that an island model can provide the necessary diversity as long as migration on a complete migration topology does not occur too frequently (or too rarely), and never occurs too close to a Maze phase transition—conditions which require somewhat specific knowledge of the fitness function, which may not be available for other problems.

In this paper, we investigate whether using a less dense migration topology, such as a unidirectional ring, can be beneficial on a dynamic problem like Maze, allowing some of the requirements on when migration is allowed to occur to be relaxed. The Maze construction requires an EA to keep an individual that is sometimes sub-optimal in the population in order to efficiently handle phase transitions; thus, maintaining population diversity is a desirable property for an island model on this function. Intuitively, decreasing the density of the migration topology weakens the negative effect of migration on population diversity, and may allow the desirable solution to survive migration occurring at inopportune times. Therefore, it is interesting to study whether this intuition can be supported by rigorous proofs. To come up with such proofs, it is necessary to present a well-defined example where the choice of the topology has a crucial impact on the optimization process. While our example will clearly support the intuition described above, it is still challenging to carry out a proof due to the amount and complexity of interaction and stochasticity in both algorithm and island model.

We have based our analysis on a simplified version of the island model studied in [17], which incorporates the major elements of the original setting: an oscillating fitness function, islands performing independent mutation/selection steps, and the effect of Maze phase transitions on the islands’ ability to track the optimum based on their current-best individuals at the time of the transition. The simplified model incorporates more randomization, as both the oscillating pattern and migration are randomized, which both simplifies the analysis, and disallows some of the more artificial solutions possible in the original model, such as only performing migrations on iterations that assign a higher fitness value to the desirable solution.

Using this simplified model, we use rigorous analysis to prove that the unidirectional ring migration topology allows the island model to track the optimum of the dynamic fitness functions in some settings where the complete migration topology and all other topologies of less than logarithmic diameter do not. We also present a converse result which applies if migration does not occur frequently enough.

This paper is structured as follows. In the next section, we introduce the simplified island model, highlighting its key differences by comparing it to the setting of [17], and introduce some of the tools used in subsequent proofs. Sections 3.1 and 3.2 consider the case of migration occurring in every iteration, the former proving that a complete migration topology as well as any topology of diameter less than $c\ln n$ for a sufficiently small constant $c>0$, leads to a failure to track the optimum, while the latter proves that switching to the unidirectional ring topology of diameter $c'\ln n$ for a sufficiently large constant $c'>0$ allows tracking the optimum with high probability. Hence, there is a sharp threshold for the topology’s diameter under which no efficient tracking is possible. Experimental results for the ring topology investigate the diversity of the population in the setting of frequent migration.

Sections 4.1 and 4.2 consider the effects of very infrequent migrations, proving that in such settings, a denser migration topology may aid in tracking the oscillating optimum. The positive result for the unidirectional ring is finally extended to the case of moderately frequent migration (instead of occurring in every iteration) in Sect. 5. We finish with some conclusions, as well as a discussion of further possibilities for analysis.

## Preliminaries

The Maze fitness function, defined formally below, and summarized in Table 1, was introduced in [11]. It is an artificial real-valued fitness function consisting of $n+1$ phases of $t_0 = kn^3$ iterations each (where $k > 0$ is a constant). Over the course of these phases the optimum shifts from the all-ones bit string, which is the optimum up to time $t_0$, to the all-zeros bit string, which is the optimum after time $(n+1)t_0$, while the majority of the search space still points towards the local optimum at the all-ones bit string. Within each phase, the optimum oscillates frequently between two specific solutions, which we will denote by $\mathrm{OPT}_p$ and $\mathrm{ALT}_p$ for each phase *p*. These solutions maintain a higher fitness value than the rest of the search points throughout the phase. Notably, OPT$_p = \mathrm {ALT}_{p+1}$, and so if an island has the OPT$_p$ individual during a phase transition, it is able to maintain a solution with a higher fitness value compared to the rest of the search space, and will not accept mutations taking it back to the all-ones bit string.

**Table 1:** The Maze dynamic fitness function: in *n* oscillating phases after an initial OneMax phase, two bit strings, $$ OPT _p$$ and $$ ALT _p$$ are assigned higher-than-OneMax fitness values

Phase	0	1	2	3	...	$n-1$	*n*	$>n$
OPT$_p$	($1^n$)	$0^{1}1^{n-1}$	$0^{2}1^{n-2}$	$0^{3}1^{n-3}$	...	$0^{n-1}1$	$0^{n}$	($0^n$)
ALT$_p$		$1^{n}$	$0^{1}1^{n-1}$	$0^{2}1^{n-2}$	...	$0^{n-2}1^2$	$0^{n-1}1^1$	

The optimum oscillates in an $$ OPT _p$$–$$ OPT _p$$–$$ ALT _p$$ pattern, with $$f( OPT _p) > f( ALT _p)$$ two iterations out of three. After phase *n*, only $$0^n$$ has a higher-than-OneMax fitness value

Formally, the Maze fitness function, defined for $x \in \{0, 1\}^n$ and $t \in \mathbb {N}_0$, is:where $\textsc {OneMax}(x) = \sum _{i=1}^n x_i$, i.e. the number of bits set to 1, and *t* is the time at which the evaluation occurs.

We note that the function was used in [11] to show that ant colony-based algorithms can be preferable to evolutionary algorithms such as the (1 $+$ 1) EA on dynamic problems, as the rapid oscillation between pairs of similar optima in each phase can more easily be represented using a pheromone memory (versus the single ancestor individual of a (1 $+$ 1) EA). While the Maze function and the simplified model that we will analyze are artificial constructions, similar effects may occur in real-world problems: noisy fitness functions might provide uncertain information about which of two good solutions is better, which can cause oscillation between the two solutions; while changing environment conditions can be similar to the phase transitions of the Maze slowly moving the global optimum through the search space.

In order to analyze the impact of the migration topology on the island model behavior, and remove some of the artifacts arising from the Maze fitness function (such as the ability to recover the oscillating optimum via a few unlikely mutations following a phase transition where no island has the OPT individual), we will construct a somewhat simplified model of the optimization algorithm, while maintaining similarities to $\lambda$ islands using (1 $+$ 1) EAs to optimize Maze. The simplified model is shown as Algorithm 1 below and explained in the following.

Some changes have been made to the model of the Maze fitness function. In Algorithm 1, islands can be in one of three states, OPT, ALT, and LOST, with each iteration randomly selecting which of OPT and ALT has a higher fitness value, favoring OPT over ALT independently probability $p_\mathrm {OPT}$. When a Maze phase transition occurs, all islands in the OPT state transition to the ALT state, while all other islands transition to the LOST state, regardless of which state was favored during the iteration. The OPT, ALT, and LOST states correspond to having OPT, ALT, and OneMax-valued individuals in the original Maze, where the OPT individual in each phase becomes the ALT individual of the next phase, while the ALT individual becomes a OneMax-valued individual following a phase transition, even if the ALT individual was assigned a higher fitness value in the iteration immediately before the phase transition.

We now elaborate on the island model in more detail. Each island *i* behaves like a simplified (1 $+$ 1) EA, maintaining a current-best solution $x^*_i(t)$ by applying mutation and selection.

The mutation operator is simplified as specified below:

$$\begin{aligned} \mathrm {mutate}(x) = \left\{ \begin{array}{ll} \text {ALT} &{}\quad \text {with probability }p_\mathrm {mut} \text { if } x = OPT \\ \text {OPT} &{}\quad \text {with probability }p_\mathrm {mut} \text { if }x = ALT \\ x &{}\quad \text {otherwise} \end{array} \right. \end{aligned}$$

which essentially prevents an island in the LOST state from mutating to OPT or ALT. Notably, the selection operator on each island compares the fitness of the mutated offspring to that of its ancestor, so only mutations that improve the current solution would be accepted.

With an appropriate choice of $p_\mathrm {mut}$ based on a probability of a specific single-bit mutation occurring, this choice of mutation and selection operators is a pessimistic model of the (1 $+$ 1) EA’s behavior on Maze, where, in the later phases, beginning a phase with a OneMax-valued individual (i. e., in the LOST state in the simplified model) would cause the (1 $+$ 1) EA to revert to optimizing OneMax with at least constant probability, leaving it with an overwhelmingly small probability of finding the oscillating optimum again [11].

Additionally, migration is randomized by allowing it to occur in each iteration independently at random with probability $p_\mathrm {mig}$. This, as well as the randomization of which of ALT and OPT is favored in any given iteration, prevents migration policies from being able to only perform migration during OPT-favoring iterations.
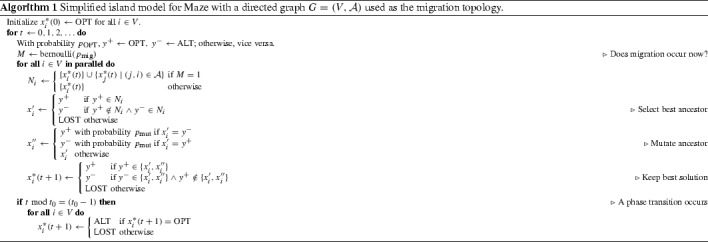


Summarized concisely, Algorithm 1 models $\lambda$ islands performing mutation in every iteration, each one updating its current-best individual $x^*_i(t+1)$ while the dynamic fitness function oscillates randomly assigning higher fitness values to OPT and ALT. When migration occurs, each island receives the previous iteration’s current-best individuals from all of the islands that are connected to it by a directed edge in the migration topology $G = (V, \mathcal {A})$, and uses the best of these individuals and its own $x^*_i(t)$ as the ancestor for the current iteration (evaluating all candidates according to the current iteration). Thus, the parameters of the simplified model are:

- *n*, the number of phases being considered,
- $t_0$, the number of iterations in each phase,
- $p_\mathrm {OPT}$, the probability of OPT having a higher fitness value than ALT in an iteration,
- $p_\mathrm {mut}$, the probability of constructing OPT from ALT and vice versa,
- $p_\mathrm {mig}$, the probability of migration occurring in an iteration,
- $\lambda$, the number of islands,
- $G = (V, \mathcal {A})$, a directed graph specifying the migration topology, with *V* being the set of vertices (islands, and therefore $|V| = \lambda$), and $\mathcal {A}$ a set of directed arcs specifying how migration transfers current-best individuals.

In this paper, we consider the impact of the migration topology *G* on the algorithm’s ability to track the oscillating OPT/ALT optimum (more specifically, whether at least one island remains in a non-LOST state after *n* oscillating phases).

The following choice of parameters yields a setting similar to the original Maze considered in [11, 17]: $t_0 = n^3$, $p_\mathrm {OPT} = 2/3$, $\lambda = \varOmega (\log n)$, $p_\mathrm {mut} = \varTheta (1/n)$, $p_\mathrm {mig} = 1/\tau$ (where $\tau$ is the deterministic migration interval), and $G = K_\lambda$. In [11], $t_0 = kn^3$ for a constant $k>0$ is used to provide an ant colony-based algorithm with sufficient time to adjust its pheromone memory. We somewhat relax the conditions on the parameter $t_0$ in our results, requiring most often that it is in $\varOmega (n^2)$ and polynomial with respect to *n*. Generally, using a constant $1/2< p_\mathrm {OPT} < 1$ allows the ant colony to adjust to the solution useful in the next iteration.

It is worth noting that in the original Maze setting, *n* serves as both the number of bits the individuals are composed of, and the number of oscillating phases in the Maze function. This motivates the relationship between *n*, $p_\mathrm {mut}$, and $\lambda$ which persists even in the simplified setting: although the simplified setting no longer deals with *n*-bit strings directly, it is still serving as a model for islands using actual (1 $+$ 1) EAs and hence also the standard bitwise mutation operator on *n*-bit strings.

To derive our theoretical results, we use the following drift theorem, which describes the expectation of the first-hitting time of a process in the presence of additive drift.

### Theorem 1

(Additive drift, expected time [7, 13]) Let $(X^t)_{t\ge 0}$, be a stochastic process over a bounded state space $S\subseteq {\mathbb {R}}_0^+$, and $\mathcal {F}_t$ a filtration to which the stochastic process is adapted (e.g., the natural filtration) and let $T_0:=\min \{t\ge 0:X^t=0\}$ denote the first hitting time of 0 and assume that both $\mathord {E }\mathord {\left( X^0\right) }$ and $\mathord {E }\mathord {\left( T_0 \mid X^0\right) }$ are finite. Then, if

$$\begin{aligned} \mathord {E }\mathord {\left( X^t-X^{t+1} \mid \mathcal {F}_t; X^t > 0 \right) } \ge \epsilon , \end{aligned}$$

it holds that $\mathord {E }\mathord {\left( T_0\mid X^0\right) } \le X^0 / \epsilon$.

To bound the probability of large deviations, the following theorem dealing with tail bounds on sums of geometrically distributed random variables is useful.

### Theorem 2

(Theorem 1.14 in [4]) Let $p\in \mathopen {]}0,1\mathclose {[}$. Let $X_1,\dots ,X_n$ be independent geometric random variables with $\Pr (X_i=j)=(1-p)^{j-1}p$ for all $j\in {\mathbb {N}}$ and let $X:=\sum _{i=1}^n X_i$.

Then for all $\delta >0$

$$\begin{aligned} \Pr (X\ge (1+\delta ) E(X)) \le e^{-\frac{\delta ^2}{2} \frac{ n-1 }{1+\delta }} \end{aligned}$$

Additionally, the classical gambler’s ruin problem [6] is used to bound the probability that a process that shrinks in expectation grows to a particular size in Lemma 7. In the canonical setting, this would be equivalent to determining the probability that a gambler who starts with a single coin is able to collect a certain number of coins in an unfair coin flipping game, e. g., where he is more likely to lose a coin than win a coin in each round.

### Theorem 3

([6], p. 345) Consider an unfair coin flipping game, where in every round, independently of previous rounds, $p\ne 1/2$ is the probability of winning one coin and $q = 1-p$ is the probability of losing a coin. Starting with *a* coins, the probability of reaching $n > a$ before reaching 0 coins equals

$$\begin{aligned} \frac{(q/p)^a-1}{(q/p)^n-1}. \end{aligned}$$

### Notation

We denote by $\log x$ the binary logarithm of *x* and by $\ln x$ the natural logarithm of *x*. If the logarithm is multiplied by an unknown constant, which is equivalent to an unknown base of the logarithm, we prefer to write $\log x$, e. g., $O(\log x)$ and $c\log x$.

We say that an event *E* occurs *with high probability* (with respect to the problem size *n*) if, for some constant $c > 0$, $P(E)=1 - O(n^{-c})$.

## Frequent Migration

As a simple case, consider setting $p_\mathrm {mig} = 1$, i. e., requiring migration to occur in every iteration. We consider two types of topologies: topologies with small diameter up to $c_1\log n$ for some sufficiently small constant $c_1>0$, and an example of a topology with diameter $\lambda =c_2 \log n$ for a sufficiently large constant $c_2>0$, namely a $\lambda$-vertex unidirectional ring. Together, these results show that the topology’s diameter is crucial for the island model to track the optimum. In fact, we prove that there a sharp threshold behavior in the domain $\varTheta (\log n)$ w. r. t. the diameter values allowing efficient tracking of the optimum.

### Topologies with Small Diameter

We first prove that using a small-diameter topology with migration occurring in every iteration results in the simplified model being unable to track the optimum of the Maze through all *n* phases. The types of topologies considered here include dense graphs such as the extreme case of complete graphs (the special case analyzed in our preliminary work [16]) but also very sparse graphs such as a star graph.

#### Theorem 4

When $t_0 \in \varOmega (n) \cap O(\mathrm {poly}(n))$, $0< p_\mathrm {OPT} < 1$ is a constant, $p_\mathrm {mut} = 1/(en)$, $p_\mathrm {mig} = 1$, $\lambda = O(n)$, and *G* is $\lambda$-vertex connected graph of diameter at most $c_1\log n$ for a sufficiently small constant $c_1\log n$, the probability that all islands are in the LOST state after $n\cdot t_0$ iterations is $1 - 2^{-\varOmega (n^{1-\epsilon })}$. Here $\epsilon =\epsilon (c_1)$ is a positive constant that can be made arbitrarily small if $c_1$ is chosen appropriately.

#### Proof

Let *k* denote the diameter of the graph. We note that between every pair of vertices there is a path of length at most *k* since the graph is assumed to be connected. The proof will analyze the probability of the ALT state spreading through the whole graph in a sequence of *k* iterations. That is, assuming migration to occur in every of the *k* iterations and ALT being the optimum, we consider the event that an ALT state residing at some vertex reaches all vertices of distance *i* within the first *i* of these iterations such that inductively all vertices of distance $i+1$ are reached within iteration $i+1$.

We note that at least one mutation occurs during a phase with at least a constant probability:

$$\begin{aligned} 1 - (1 - p_\mathrm {mut})^{\lambda t_0} \ge 1 - e^{-c}, \end{aligned}$$

and the probability that no mutation occurs in a single iteration is also at least a constant:

$$\begin{aligned} (1 - p_\mathrm {mut})^{\lambda } \ge e^{-c'}, \end{aligned}$$

where $c > 0$ and $c' > 0$ are constants.

Thus, with at least probability $(1 - e^{-c})e^{-c'k} \ge e^{-c'' c_1 \log n}$ for some constant $c''>0$, the last mutation in a phase occurs at least $k+1$ iterations before the phase transition. With probability $(1-p_\mathrm {OPT})^{k+2}\ge e^{-c''' c_1 \log n}$, for some constant $c'''>0$, both the iteration when the last mutation occurs, and all the iterations immediately following it favor ALT over OPT; thus, if all islands were in the OPT state, the mutation would produce an ALT individual which would migrate to all islands in the subsequent *k* iterations, while if at least one island was in the ALT state, its original individual would migrate to all other islands in the subsequent *k* iterations. As no further mutation occurs before the phase transition, we conclude that each phase has at least a probability $e^{-(c''+c''') c_1 \log n}$ of ending with all islands having the ALT individual, and thus losing track of the oscillating optimum following the next phase transition.

Thus, if each of *n* phases has at least a probability of failing $e^{-(c'+c'') c_1 \log n}$, the probability that at least one of *n* phases ends with all islands in the LOST state is at least $1 - (1-e^{-(c+c') c_1 \log n})^n = 1 - 2^{-\varOmega (n^{1-\epsilon })}$ if the constant $c_1$ is chosen small enough. $\square$

It is worth noting that this proof approach is flexible enough to be adapted to settings where migration occurs less often, such as once in every constant number of iterations. The proof of Theorem 4 essentially relies on no mutations occurring and ALT being preferred throughout a sequence of $k+1$ migrating steps before the phase transition. Suppose $p_\mathrm {OPT}$ is at most a constant smaller than 1, and $c'k$ steps, for a sufficiently large constant $c'>0$, contain at least *k* migrations. Then, with probability $e^{-c''k}$ for a sufficiently large constant $c''>0$ (depending on $c'$ and $p_\mathrm {OPT}$), migration propagates the ALT state to all islands, and the model becomes LOST in the subsequent phase transition. Choosing the implicit constant in *k* small enough, we arrive again at a probability of $1 - 2^{-\varOmega (n^{1-\epsilon })}$ of losing track of the optimum.

### Unidirectional Ring Topology with Sufficiently Large Diameter

We suppose now that *G* has a sufficiently large diameter by being minimally connected, i. e., *G* is a unidirectional ring of $\lambda$ vertices and $\lambda$ arcs. This reduces the effect of migration on the island memory, making it impossible to propagate an undesirable individual to all islands in a single migration. In this section, we will prove that the simplified island model is able to track the oscillating optimum for the full *n* phases.

#### Theorem 5

When $$t_0 \in \varOmega \!\left( n^2\right) \cap O(\mathrm {poly}(n))$$, $$p_\mathrm {OPT} = 1/2+\epsilon $$ for some constant $$\epsilon >0$$, $$p_\mathrm {mut} = 1/(en)$$, $$p_\mathrm {mig} = 1$$, $$\lambda = c\log n$$, where $$c > 0 $$ is a sufficiently large constant, and *G* is a $$\lambda $$-vertex unidirectional ring, the simplified island model is able to track the oscillating optimum for at least *n* phases with high probability.

We will prove this by showing that as long as each phase begins with at least one island still tracking the optimum, the phase will end with at least one island *i* having $x^*_i(t) = \text {OPT}$. Roughly speaking, Lemma 6 first proves the number of OPT-islands to grow to $\lambda$ within a phase, whereafter Lemma 7 states that this number does not drop to 0 in the remainder of the phase, both with high probability.

Notably, for the results any constant $p_\mathrm {OPT} > 1/2$ is sufficient, including the choice $p_\mathrm {OPT} = 2/3$ corresponding to the oscillation pattern of the original Maze.

#### Lemma 6

Let, as in the setting of Theorem 5, $$t_0 \in \varOmega \left( n^2\right) \cap O(\mathrm {poly}(n))$$, $$p_\mathrm {OPT} = 1/2+\epsilon $$ for some constant $$\epsilon >0$$, $$p_\mathrm {mut} = 1/(en)$$, $$p_\mathrm {mig} = 1$$, $$\lambda = c\log n$$, where $$c > 0 $$ is a sufficiently large constant, and *G* be a $$\lambda $$-vertex unidirectional ring. If a phase begins with at least one island *i* having $$x^*_i(t') \ne \mathrm {LOST}$$, there will with high probability exist an iteration $$t'' \ge t'$$ before the phase ends such that all islands will have $$x^*_i(t'') = \mathrm {OPT}$$.

#### Proof

We note that after at most $\lambda$ iterations, no islands will be in the LOST state, as $\lambda$ iterations are enough to migrate the non-LOST individual from any surviving island to all other islands, with fewer iterations being required if there is more than one surviving island.

Let $t'$ be the iteration during which no islands are in the LOST state, and consider the drift in $X_t$, the number of islands *i* having $x^*_i(t+t') = \mathrm {ALT}$. Let $S_t$ be the number of arcs $(u,v) \in \mathcal {A}$ in the migration topology for which it holds that $x^*_u(t+t') = \mathrm {OPT}$ and $x^*_v(t+t') = \mathrm {ALT}$, i. e., the number of segments in the unidirectional ring of the migration topology which are composed of islands having OPT as their current-best solution. We note that, regardless of which solution is favored when a migration occurs, $S_t$ islands will change their current-best solution because of migration—there are $S_t$ segments of OPT islands in the migration topology; the first island in each segment will receive ALT through migration, while the islands following each segment will receive OPT through migration, so regardless of which solution is favored in an iteration, migration will cause $S_t$ islands to switch state. The remaining islands, if they are in the non-favored state, may change their current-best solution through mutation. The expected change in $X_t$ is a combination of these effects:where the inequalities reflect lower-bounding the positive contribution of mutation as 0, upper-bounding $(\lambda - X_t - S_t) < \lambda$ in the negative contribution of mutation, and recalling that $p_\mathrm {mig} = 1$, $2 p_\mathrm {OPT} > 1$ and $\lambda \in o(1/p_\mathrm {mut})$.

When the iteration begins with no islands in the OPT or LOST states, a drift toward $X_t = 0$ exists,

$$\begin{aligned} E(X_{t} - X_{t+1} \mid X_t = \lambda ) = p_\mathrm {OPT} \, p_\mathrm {mut} \, \lambda = \frac{2 c \log n}{3en}, \end{aligned}$$

and can be used as a lower bound on the overall drift throughout the whole process.

Applying the additive drift theorem, the expected first hitting time $T = \min \{t{:}\,X_t = 0\} = O(\lambda /\tfrac{2c \log n}{3en}) = O(n)$. As this is much shorter than the phase length $t_0 \in \varOmega (n^2)$, we can conclude that $X_t = 0$ is hit during the phase with high probability (by applying a Markov bound on the probability that the first hitting time exceeds twice the expectation, and repeating the argument *n* times), and hence at least at some point during the phase, all islands have OPT as their current-best solution. $\square$

We now need to show that it is not likely that the island model will manage to replace OPT with ALT on all islands during the remainder of the current phase.

#### Lemma 7

Let $$t_0 \in \varOmega \!\left( n^2\right) \cap O(\mathrm {poly}(n))$$, $$p_\mathrm {OPT} = 1/2+\epsilon $$ for some constant $$\epsilon >0$$, $$p_\mathrm {mut} = 1/(en)$$, $$p_\mathrm {mig} = 1$$, $$\lambda = c\log n$$, where $$c > 0 $$ is a sufficiently large constant, and *G* be a $$\lambda $$-vertex unidirectional ring. If there occurs an iteration where $$x^*_i(t) = \mathrm {OPT}$$ for all islands, then, with high probability, at least one island will be in a non-LOST state following the next phase transition.

#### Proof

We note that it is difficult to apply a negative drift theorem directly in this setting, as the drift would depend on $S_t$: if there are many OPT/ALT boundaries in the migration topology, migration may cause drastic changes in the number of islands having OPT as their current-best individual. Instead, our strategy is to bound the number of islands having ALT as their current-best individual by considering the effects of each OPT-to-ALT mutation that occurs in isolation, i. e., as if it created the only ALT segment around at any specific time. An upper bound on the total number of islands having ALT as their current-best solution at any specific time can then be derived from bounds on the maximum length each isolated ALT segment may reach, the number of iterations isolated ALT segments survive, and the rate at which such segments are created.

When considered in isolation, an OPT-to-ALT mutation creates an ALT segment with initial length 1 in the migration topology. We only consider its length to be modified by migration: it increases by 1 if migration occurs during an ALT-favoring iteration, and decreases by 1 if migration occurs during an OPT-favoring iteration; any further OPT-to-ALT mutations would be treated as separate isolated segments, and pessimistically, no ALT-to-OPT mutations occur within the ALT segment. There is a tendency towards decreasing the length. Instead of a drift theorem, here we even can directly apply results on the classical gambler’s ruin problem (Theorem 3) to bound the maximum length of such an isolated segment: it decreases with probability at least $1/2+\epsilon$, as $p_\mathrm {OPT} = 1/2+\epsilon$, the maximal possible change is by 1 in either direction, and the probability of increasing is at most $1/2-\epsilon$. Let $r:=(1/2+\epsilon )/(1/2-\epsilon )$ and note that *r* is a constant greater than 1. Thus, using the ruin problem on $\{0,\dots ,\ell \}$ with starting state 1, the probability that the length of an ALT segment exceeds $\ell = c'\log n$, where $c'>0$ is constant is no more than

1 $$\begin{aligned} \frac{r^1-1}{r^{\ell }-1 } \le r^{-\ell +1} = O( n^{-c'}). \end{aligned}$$

Next, consider the number of iterations before migration reduces a freshly-created ALT segment to length 0. This can be upper-bounded using a tail bound on the binomial distribution: the segment is guaranteed to be reduced to length 0 if, in 2*k* iterations, at least *k* favor OPT. Let $X_{2k}$ be the number of iterations that favor OPT of 2*k* iterations:

$$\begin{aligned} P(X_{2k} \le k) \le \mathrm {exp}\left( -\frac{2k(1/2+\epsilon )}{3} \left( \frac{\epsilon }{1+\epsilon }\right) ^2\right) = e^{-\varOmega (k)} \end{aligned}$$

using Chernoff’s inequality with $\delta =\epsilon /(1+\epsilon )$ and recalling $p_\mathrm {OPT} = 1/2+\epsilon$. Setting also $k = c'\log n$ for large enough but constant $c'>0$, we conclude that, with probability $1 - n^{-\varOmega (c')}$, an OPT-to-ALT mutation disappears after $O(\log n)$ iterations. In total, the expected number of OPT-to-ALT mutations within a phase is at most $(1-p_\mathrm {OPT}) \,p_\mathrm {mut} \, \lambda \, t_0 = O\!\left( t_0\log (n)/n\right)$ since $p_\mathrm {mut} = 1/(ne)$, so by a straightforward union bound on the probabilities of an ALT segment surviving for longer than $O(\log n)$ iterations, none of the OPT-to-ALT mutations that occur in the considered interval survive for more than the desired number of iterations with high probability. Here we assume that $c'$ is chosen large enough that the total failure probability in the phase of $t_0$ steps is smaller than any given probability $1/n^d$ for some constant $d>0$.

Finally, we need to show that the rate at which OPT-to-ALT mutations are accepted is low enough to allow any accepted mutations to dissolve through migration without overrunning the island model. To that end, we can bound $Y_k$, the number of OPT-to-ALT mutations that are accepted within $k = c'\log n$ iterations using a Chernoff bound:

$$\begin{aligned}&E(Y_k) < k \, \lambda \, p_\mathrm {mut} = O((\log ^2 n)/n) = o(1) \\&P(Y_k \ge c' \ln n) \le e^{-c'\ln n} = n^{-c'} \end{aligned}$$

recalling that $\lambda = O(\log n)$, $p_\mathrm {mut} = 1/(ne)$, and ignoring the possibility that some of these mutations occur during iterations which assign a higher fitness value to OPT, and therefore would not be accepted.

**Fig. 1:**
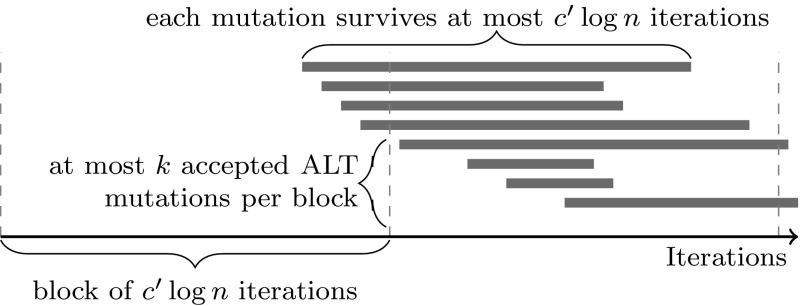
Lemma 7: the oscillating phase is divided into blocks of $c' \log n$ iterations; with high probability, no more than $k = c' \ln n$ OPT-to-ALT mutations are accepted within each block, each one creating an ALT segment that disappears after at most $c' \log n$ iterations with high probability. Thus, with high probability, at most 2*k* ALT segments may be active at the same time

Thus, no more than $c'\ln n$ OPT-to-ALT mutations are accepted during a $c'\log n$ iteration period with high probability, and all accepted mutations disappear after $c'\log n$ iterations with high probability. By dividing the Maze phase into blocks of $c'\log n$ iterations each, as illustrated in Fig. 1, we can conclude that with high probability, at most $2 \cdot c'\log n = O(\log n)$ OPT-to-ALT segments can be active at the same time: with high probability, no more than $c' \log n$ appear at the exact end of an $c'\log n$ iteration block, and no more than $c'\log n$ appear during the next block, with the former group all being reduced to length 0 before the next-next block begins.

We are finally ready to bound the total number of islands that can have ALT as their best-so-far individual at the same time: denoting by *s* the number of segments consisting of ALT-individuals, we define $L_i$ as the length of the *i*th segment. We are interested in $S:=\sum _{i=1}^s L_i$, which is the total number of ALT-islands. By (1), we have $P(L_i\ge j)\le r^{-j+1}$, independently from the other segments. Hence, $L_i-1$ is stochastically dominated by a geometrically distributed random variable with parameter 1 / *r* and $S-s$ is dominated by the sum of *s* such random variables. We assume $s\le 2c'\log n$, which, as argued before, holds with high probability. Now we can apply Theorem 2 on the sum of geometric random variables, choosing $\delta =3$, and get that $P(S\ge s+(8c'\log n)/r) \le e^{-\frac{9(2c'\log n-1)}{8}} \le n^{-c'}$. Altogether, for a sufficiently large *n* and a sufficiently large constant *c* from the lemma, there will with high probability still be an island with $x^*_i(t) = \mathrm {OPT}$ at the end of the phase, and hence will be in a non-LOST state following the phase transition. $\square$

We note that the bounds used in Lemma 7 take a very dim view of the situation, and could probably be improved significantly. In practical simulations, such as the experiments presented in Sect. 3.3, we observe that the simplified island model converges to a larger-than-$p_\mathrm {OPT}$ majority of islands having OPT as their current-best solution, and any OPT-to-ALT mutations disappear quickly.

Applying Lemmas 6 and 7 inductively over *n* phases yields a proof of Theorem 5.

#### Proof (of Theorem 5)

For the first iteration, Lemma 7 may be applied immediately, as all islands are initialized with the OPT individual. Per the lemma, at least one island *i* ends the phase with $x^*_i(t) = \mathrm {OPT}$ with high probability, allowing Lemma 6 to be applied at the beginning of the next phase. Per that lemma, there is with high probability an iteration within the phase when OPT is the current-best individual on all islands, allowing Lemma 7 to be applied again.

As the events described in both of these lemmas occur with high probability, and we only require *n* repeated applications of each lemma to cover the whole optimization process, a simple union bound on the failure probabilities can be used to conclude that with high probability, at least one island is still tracking the oscillating optimum after the *n* phases are over. $\square$

Thus, we have proven that using a unidirectional ring of diameter $c\ln n$ for sufficiently large constant $c>0$ as the migration topology can allow the simplified island model to track the oscillating optimum of the Maze in settings where this is not possible for the complete migration topology. Intuitively, this is achieved by removing the ability of a single ill-timed migration to propagate an undesirable individual to all islands. Together with the result from Sect. 3.1, we have determined a sharp threshold around $\varTheta (\log n)$ for the diameter of the topology which is necessary to track the optimum.

### Experimental Results

While Theorem 5 proves that constant migration on a sufficiently-large ring topology can track the optimum of the Maze through *n* phase transitions by showing that, with high probability, there is at least one island in the OPT state at the end of a phase, it does not provide an upper bound on the expected number of islands in the OPT state at the end of the phase, and requires $p_\mathrm {OPT} > 1/2$ for its proof. This condition on $p_\mathrm {OPT}$ is used in Lemmas 6 and 7 to show that there is a drift towards recovering OPT islands after a phase transition, and any OPT-to-ALT mutations are quickly undone.

An interesting question to consider experimentally is whether the combination of constant migration and a ring migration topology is an effective diversity-preserving mechanism. If the islands were to split between OPT and ALT states according to $p_\mathrm {OPT}$, it might also be possible to track the optimum also for a constant $0 < p_\mathrm {OPT} \le 1/2$. If, on the other hand, these migration parameters only ensure that the simplified island model detects that $p_\mathrm {OPT} > 1/2$, and keeps a far greater number of islands in the OPT state, tracking the optimum for smaller $p_\mathrm {OPT}$ values would likely be impossible.

We examine this issue experimentally, by simulating the simplified island model following a particularly bad phase transition, with only a single island surviving in the ALT state. 1000 independent simulations of this setting are performed, with $\lambda = 100$ islands and $p_\mathrm {mut} = 1/2000$ chosen to model the typical relationship between $\lambda = c \log n$ islands and $p_\mathrm {mut} = 1/(en)$ probability of transitioning between the states via mutation, making state transitions due to mutation relatively rare, while maintaining a reasonable time limit on the number of iterations to simulate. We note that we stop the simulation after 2000 iterations (with no phase transition occurring); a typical choice of $t_0 = n^3$ would require significantly more iterations.

**Fig. 2:**
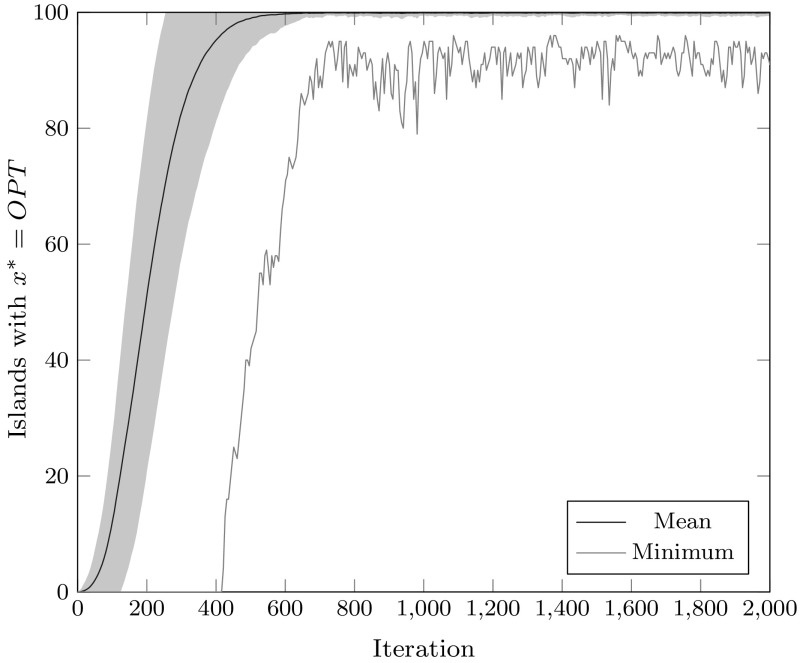
1000 simulations of an island system with $\lambda = 100$ islands running the simplified island model, using a unidirectional ring as the migration topology, and $p_\mathrm {mig} = 1, p_\mathrm {OPT} = 2/3, p_\mathrm {mut} = 1/2000$, initialized with a single island in the ALT state at iteration 1, and all remaining islands in the LOST state. The plot shows the average (mean) number of islands in the OPT state, standard deviation from this mean (shaded blue region), and the minimum observed number of OPT islands at a given iteration across the 1000 simulations (Color figure online)

**Fig. 3:**
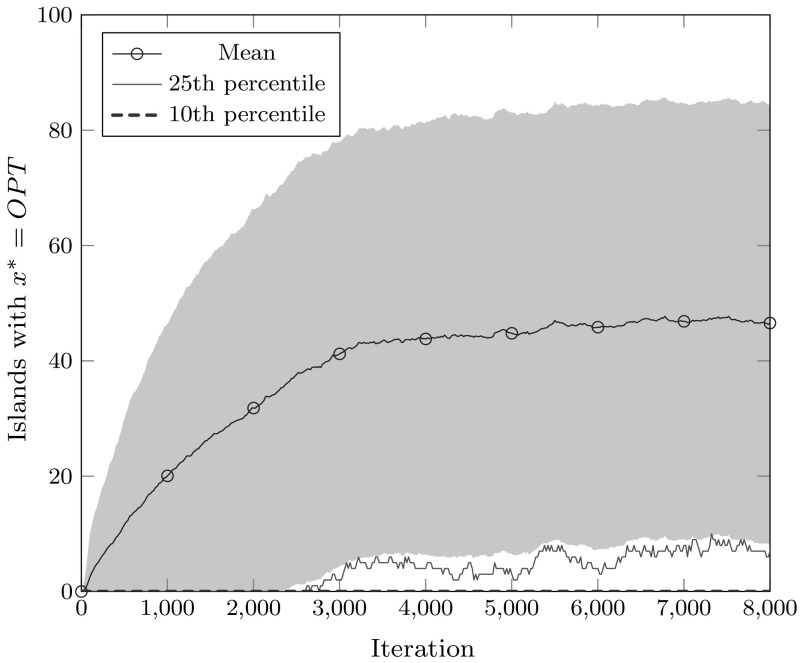
1000 simulations of an island system with $\lambda = 100$ islands running the simplified island model, using a unidirectional ring as the migration topology, and $p_\mathrm {mig} = 1, p_\mathrm {OPT} = 1/2, p_\mathrm {mut} = 1/2000$, initialized with a single island in the ALT state at iteration 1, and all remaining islands in the LOST state. The plot shows the average (mean) number of islands in the OPT state, standard deviation from this mean (shaded blue region), as well as the 25th and 10th percentiles of the observed number of OPT islands at a given iteration across the simulations (Color figure online)

The results are shown in Fig. 2. With $\lambda = 100, p_\mathrm {mut} = 1/2000$, and $p_\mathrm {mig} = 1$, the simplified island model appears to reach a steady state less than 1000 iterations after the simulated phase transition, with an average of 99.91 islands in the OPT state, and an observed standard deviation of around 0.60; similarly, after 1000 iterations have elapsed, the worst of the 1000 simulations always has at least 82 islands in the OPT state, with an average of around 92.

Overall, the simulation suggests that constant migration using a ring topology will in expectation result in the island model converging to the favored optimum, rather than maintaining an equilibrium close to $p_\mathrm {OPT}$. This suggests that when $p_\mathrm {OPT} < 1/2$, this choice of migration parameters will not be able to reliably track the Maze optimum. This is illustrated in the experimental results presented in Fig. 3, which shows the same setting with $p_\mathrm {OPT} = 1/2$ simulated for 8000 iterations following the phase transition: the variance on the number of islands in the OPT state remains high, implying that instead of having the simulations converge on having an approximately even split of islands between OPT and ALT states, the simulations alternate between having a large majority of the islands in the OPT state and having a large majority of the islands in the ALT state.

## Occasional Migration

In this section, we consider the behavior of the island model when migration occurs less frequently. In particular, we demonstrate that with $p_\mathrm {mig} = O(1/t_0)$, the ring topology is not able to track the optimum through *n* phases, while the complete migration topology with the same migration frequency is able to do so.

The following lemma provides a useful bound on the distribution of the non-LOST island states immediately prior to a phase transition in cases where migration does not occur close to the phase transition. Its proof follows the approach used in [17] to analyze the behavior of a single $(1+1)$ EA island on Maze.

### Lemma 8

Let $0< p_\mathrm {OPT} < 1$ be a constant, and let $0 < p_\mathrm {mut} \le 1/4$. Assuming no migration or phase transitions have occurred for at least $t = 2k/p_\mathrm {mut}$ iterations, where *k* is a large-enough constant, the probability $p_A$ that a non-LOST island is in an ALT state can be bounded by constants $a \le p_A \le b$ such that $a > 0$ and $b < 1$.

### Proof

**Fig. 4:**
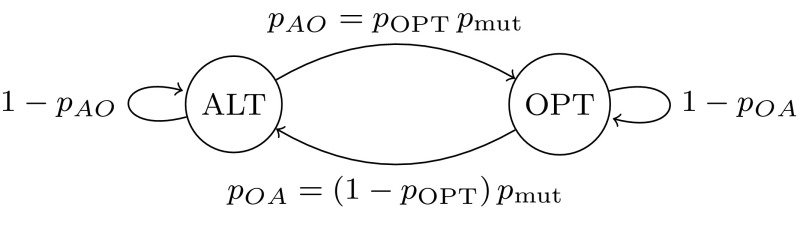
Island behavior in the absence of migration and phase transitions, modeled as a two-state Markov chain in the proof of Lemma 8

In the absence of migration, the behavior of a non-LOST island can be modeled by a Markov chain over the two states ALT and OPT (corresponding to the current-best individuals), illustrated in Fig. 4. The steps of the Markov chain correspond to iterations of the simplified island model, and the probabilities of transitioning between the states are $p_{ AO } = p_\mathrm {OPT} p_\mathrm {mut}$ and $p_{ OA } = (1-p_\mathrm {OPT})p_\mathrm {mut}$ respectively.

Let $\mathbf {\pi } = \left( \pi _\mathrm {ALT}, \pi _\mathrm {OPT}\right)$ be the steady-state distribution of the Markov chain, and consider $\pi _\mathrm {ALT}$, the probability of the chain being in the ALT state:

$$\begin{aligned} \pi _\mathrm {ALT}&= \pi _\mathrm {OPT} p_{ OA } + \pi _\mathrm {ALT} (1 - p_\mathrm {AO})\\&= \pi _\mathrm {OPT} (1-p_\mathrm {OPT}) p_\mathrm {mut} + \pi _\mathrm {ALT} (1 - p_\mathrm {OPT} p_\mathrm {mut})\\&= (1 - \pi _\mathrm {ALT})(1-p_\mathrm {OPT}) p_\mathrm {mut} + \pi _\mathrm {ALT} (1 - p_\mathrm {OPT} p_\mathrm {mut}) \\&= (1-p_\mathrm {OPT}) p_\mathrm {mut} + \pi _\mathrm {ALT} (1 - p_\mathrm {mut}), \end{aligned}$$

as $\pi _\mathrm {OPT} + \pi _\mathrm {ALT} = 1$, and by rearranging:

2 $$\begin{aligned}&\pi _\mathrm {ALT} - \pi _\mathrm {ALT} (1 - p_\mathrm {mut}) = (1-p_\mathrm {OPT}) p_\mathrm {mut} \nonumber \\&\pi _\mathrm {ALT} p_\mathrm {mut} = (1-p_\mathrm {OPT}) p_\mathrm {mut} \nonumber \\&\pi _\mathrm {ALT} = 1-p_\mathrm {OPT}. \end{aligned}$$

Over time, the distribution of the island’s state approaches the steady-state distribution of this Markov chain. To bound the total variation distance (i. e., $\tfrac{1}{2} \sum _{s\in \varOmega } |p_s - \pi _s|$, where $p_s$ is the probability that the Markov chain is in state *s* at a particular time), we use a coupling time argument: the total variation distance at time *t* is at most the probability that two independent instances of the Markov chain, started in different states, have not ever been in the same state by time *t*, as proven in e.g. [14, Theorem 5.2]. Using $p_{ OA }$ and $p_{ AO }$ to denote the transition probabilities, the total variation distance at time *t* is thus at most

$$\begin{aligned}&\left( 1 - p_{ OA } (1 - p_{ AO }) - (1 - p_{ OA }) p_{ AO }\right) ^t \\&\quad = \left( 1 - p_{ OA } - p_{ AO } + 2 \,p_{ OA }\,p_{ AO }\right) ^t \\&\quad = \left( 1-p_\mathrm {mut} + 2 {p_\mathrm {mut}}^2 (p_\mathrm {OPT}- {p_\mathrm {OPT}}^2)\right) ^t \\&\quad \le (1 - p_\mathrm {mut}/2)^t \end{aligned}$$

using that $(p_\mathrm {OPT}- {p_\mathrm {OPT}}^2) < 1$, and $2 {p_\mathrm {mut}}^2 \le p_\mathrm {mut}/2$ for $p_\mathrm {mut} \le 1/4$.

Thus the total variation distance after $t = 2k/p_\mathrm {mut}$ iterations is at most:

$$\begin{aligned} (1 - p_\mathrm {mut}/2)^{2k/p_\mathrm {mut}} \le e^{-k}, \end{aligned}$$

and therefore $p_A$, the probability that the island ends the phase with ALT as its current-best individual, can differ from $\pi _\mathrm {ALT}$ by at most $e^{-k}/2$:

$$\begin{aligned} \pi _\mathrm {ALT} - e^{-k}/2 \le p_A \le \pi _\mathrm {ALT} + e^{-k}/2, \end{aligned}$$

and, by substituting (2),

$$\begin{aligned} (1 - p_\mathrm {OPT}) - e^{-k}&\le p_A \le (1 - p_\mathrm {OPT}) + e^{-k}, \end{aligned}$$

which are constant when $p_\mathrm {OPT}$ is a constant and *k* is a large-enough constant. $\square$

### Corollary 9

When migration does not occur significantly more often than mutation, i. e., $p_\mathrm {mig} \in O(p_\mathrm {mut})$, and $0< p_\mathrm {OPT} < 1$ is a constant, the probability $p_A$ that a non-LOST island is in the ALT state $\varOmega (1/p_\mathrm {mut})$ iterations after a phase transition (or after the island becoming non-LOST), can be bounded by constants $a \le p_A \le b$, where $a > 0,b < 1$.

### Proof

The approach used to prove Lemma 8 can be adapted to this setting.

For the lower bound on $p_A$, we pessimistically assume that migration, when it occurs, always causes a transition from the ALT state to the OPT state; as $p_\mathrm {mig} \in O(p_\mathrm {mut})$, this increases $p_{ AO }$ by at most a constant factor, and hence increases $\pi _\mathrm {ALT}$ by at most a constant.

For the upper bound on $p_A$, we similarly assume that migration always causes a transition from the OPT state to the ALT state, increasing $p_{ OA }$ by at most a constant factor, and hence decreasing $\pi _\mathrm {ALT}$ by at most a constant.

Increasing the transition probabilities between states can only shorten the time required to reduce the total variation distance down to the desired level, so the $e^{-k}$ bound on total variation distance from the Markov chain steady-state distribution can be applied without further modifications. $\square$

### Ring Topology

With migration occurring an expected constant number of times in each phase, using the unidirectional ring as the migration topology results in all islands being in the LOST state at the end of *n* phases.

#### Theorem 10

When $t_0 \in \varOmega \!\left( n^2\right) \cap O(\mathrm {poly}(n))$, $1/2+\epsilon \le p_\mathrm {OPT} \le 1-\epsilon$ for some constant $\epsilon >0$, $p_\mathrm {mut} = 1/(en)$, $p_\mathrm {mig} = 1/(k t_0)$, where $k > 1$ is a large-enough constant (possibly depending on $\epsilon )$, $\lambda = O(n^{1-\epsilon })$, and *G* is a $\lambda$-vertex unidirectional ring, the simplified island model will with high probability have all islands in the LOST state by the end of phase *n*.

#### Proof

From all consecutive segments of LOST islands in the migration topology at the start of phase *p*, let *L* be the one that is longest and includes the island of lowest index. Let $X_p$ be the number of islands *not* in *L*. We would like to apply the additive drift theorem to $X_p$, showing that there exists a drift toward 0, and, as $\lambda = O(n^{1-\epsilon })$, $X_p = 0$ is hit before the *n* phases are over. This corresponds to *L* growing to maximum length.

We begin by showing a negative drift on $X_{p}$ when all islands are in a non-LOST state (i.e. $X_p = \lambda$). In this case, by Lemma 8, with probability greater than $1 - p_\mathrm {mig} \cdot 2k/p_\mathrm {mut} = 1 - O(n^{-1})$, no migration occurs too close to the phase transition, and thus all islands are within a constant variation distance of the steady-state distribution. The drift can be bounded by considering the contribution of a single island:

$$\begin{aligned} E(X_{p} - X_{p+1} \mid X_p = \lambda )&\ge (1 - p_\mathrm {OPT}- e^{-k}) \left( 1 - O(n^{-1})\right) \\&= \varTheta (1) \end{aligned}$$

since $p_\mathrm {OPT} +e^{-k}\le 1-\epsilon /2$ if *k* is large enough.

While $0< X_p < \lambda$, the LOST segment may shrink before the phase transition as migration is able to recover LOST islands, and may grow following the phase transition as some islands transition to the LOST state. Let $\delta ^-$ be the negative contribution of migration during the phase, $\delta ^+$ be the positive contribution of the phase transition, and *R* be the event that $0< X_p < \lambda$; we would like to show that:

$$\begin{aligned} E(X_{p} - X_{p+1} \mid R) \ge E(\delta ^+ \mid R) - E(\delta ^- \mid R) > c, \end{aligned}$$

where $c > 0$ is constant.

The negative contribution $\delta ^-$ can be upper-bounded as the number of migrations that occur during the $t_0$ iterations in a phase, i. e., a binomially-distributed variable:

$$\begin{aligned} E(\delta ^- \mid R) \le t_0 \, p_\mathrm {mig} = 1/k. \end{aligned}$$

The positive contribution $\delta ^+$ can be lower-bounded by focusing on the non-LOST island immediately following the LOST segment *L*. If this island is in the ALT state at the phase transition, the LOST segment length will increase by at least 1 following the phase transition. We note that for as long as the LOST segment preceding it does not disappear entirely, this island is not affected by migration, which allows the strategy used in the proof of Lemma 8 to be applied.

When this island *is not* affected by migration, the true probability of having ALT as the current-best individual approaches $\pi _\mathrm {ALT} = 1 - p_\mathrm {OPT}$ from above, as the island begins phase *p* with ALT as its current-best solution (due to the phase transition preceding phase *p*). This allows us to use $1 - p_\mathrm {OPT} \ge \epsilon$ as a lower bound on $E(\delta ^+ \mid R)$ when this island is not affected by migration.

When the island *is* affected by migration, Corollary 9 can be applied: even in the presence of migration to the considered island, the probability that it ends the phase in an ALT state, and hence $E(\delta ^+ \mid R)$, can be lower-bounded by a positive constant.

Returning to the overall drift,

$$\begin{aligned} E(X_{p} - X_{p+1} \mid R) = E(\delta ^+ \mid R) - E(\delta ^- \mid R) = \varOmega (1) - 1/k \end{aligned}$$

i. e., for a large-enough constant *k*, there is a constant drift toward $X_p = 0$.

Applying the additive drift theorem, the expected first hitting time of $X_p = 0$ is $O(\lambda ) = O(n^{1-\epsilon })$ phases. We note that the probability that this does not happen in twice the expected number of phases is, by applying Markov’s inequality, at most 0.5; and after $\varOmega (n^{\epsilon })$ repetitions, at most $2^{-\varOmega (n^{\epsilon })}$. Therefore, with high probability, the ring topology loses track of the optimum on all islands before the *n* phases are over. $\square$

This serves as an illustration that with $p_\mathrm {mig} < 1/(k \, t_0)$, where $k > 1$ is a sufficiently large constant, migration on a ring topology is not able to recover islands lost in phase transitions sufficiently quickly. In such circumstances, denser migration topologies may have an advantage, as they are able to repopulate more islands per migration, and therefore also track the optimum through a greater number of phases.

### Complete Topology

In [17], it was proven that a complete migration topology loses track of the Maze optimum if migrations occurred less frequently than once in every $O(\log (\lambda )t_0)$ iterations. This result also points to a negative result for the complete topology with $p_\mathrm {mig} \in O(1/t_0)$ in the simplified model, as the time between migrations, which is geometrically distributed, may exceed $c \; t_0 \log (n)$ iterations with probability $n^{-c/k}$, where $k > 0$ is a constant. Partitioning the optimization process into $\varOmega (n /\!\log n)$ stages (of $\varTheta (\log n)$ phase transitions each), we conclude that with migration rate $p_\mathrm {mig} = 1/(k \, t_0)$, the complete topology will fail at least one such stage with high probability, and therefore will fail to track the optimum through the *n* phases.

We note that Theorem 10 would also apply to any migration schedule with the same expected number of migrations. On the other hand, there is a randomized migration schedule, with the same expected number of migrations, for which a complete migration topology is able to track the optimum through all *n* phases even with $\lambda \in O(\log n)$ islands.

#### Theorem 11

Let $t_0 \in \varOmega \!\left( n^2\right) \cap O(\mathrm {poly}(n))$, $0<p_\mathrm {OPT} <1$ a constant, $p_\mathrm {mut} = 1/(en)$, $\lambda \in \varOmega (\log n)$, *G* be a complete $\lambda$-vertex graph, and let migration occur once every $k t_0$ iterations (where $k > 1$ is a constant), with the iteration being chosen uniformly at random. The simplified island model is able to track the optimum through *n* phases of $t_0$ iterations each with high probability.

#### Proof

We note that the maximum number of iterations between any two migrations in this schedule is $2k t_0$, corresponding to migration occurring on the first and last iterations of two adjacent $k t_0$ iteration blocks; thus, at most 2*k* phases can elapse without migration.

Consider the probability that a single island loses track of the oscillating optimum in 2*k* phase transitions: in the absence of migration, Lemma 8 applies, and the probability of a non-LOST island ending a phase with an ALT current-best individual is at most a constant smaller than 1. Thus, the probability that the island survives through 2*k* phase transitions, where *k* is a constant, is also a constant; and therefore, the probability that at least one of $\lambda = \varOmega (\log n)$ islands survives is at least $1 - n^{-c}$, where $c > 0$ is a constant.

Thus, as long as at least one island survives a migration-less period, the complete migration topology will allow all islands to recover from the LOST state. With a sufficiently large $\lambda$, the probability that at least one island survives through each of the at most *O*(*n*) migration-less periods can be made polynomially high, and hence the complete migration topology will be able to track the oscillating optimum through all *n* phases with high probability.

We note that this process relies on no migration occurring too close to a phase transition, as, in the worst case, this could migrate the ALT individual to all islands, resulting in all islands losing track of the oscillating optimum when the phase transition occurs. Per Lemma 8, this is not a problem as long as no migration occurs within $O(1/p_\mathrm {mut}) = O(n)$ iterations of each phase transition; and so we note that there are at most $O(n^2)$ iterations during which migration should not occur, and this constraint is respected with probability at least $1 - O(n^2p_\mathrm {mig}) = 1 - O(n^{-1})$. Thus, with high probability, this problematic situation does not occur. $\square$

## Moderately-Frequent Migration on the Ring

If migration on the ring topology occurs sufficiently often to recover all of the lost islands, and yet rarely enough to ensure that the distribution of the island states is governed primarily by the mixing time argument, the simplified island model may track the optimum of the Maze through *n* oscillating phases while preserving diversity in the island population, allowing the oscillating optimum to be tracked for any constant $p_\mathrm {OPT} > 0$, rather than the $p_\mathrm {OPT} > 1/2$ required by Theorem 5.

### Theorem 12

When $\lambda \ge c \log n$, where *c* is a sufficiently-large constant, $t_0 = \omega (\lambda /p_\mathrm {mig})$, $p_\mathrm {mut} = 1/(en)$, $0<p_\mathrm {OPT} <1$ a constant, the migration topology is a unidirectional ring, and $p_\mathrm {mig} = n^{-1.5}$, the probability that the simplified island model has at least one non-LOST island after $n \cdot t_0$ iterations is at least $1 - O(1/n)$.

### Proof

We note that as long as at least one island is in a non-LOST state following a phase transition, in $O(\lambda /p_\mathrm {mig})$ iterations, all islands will be in a non-LOST state with high probability. This can be shown by applying a Chernoff bound on a the number of migrations occurring within $2\lambda /p_\mathrm {mig}$ iterations: the probability that this is less than half of its $2\lambda$ expectation is at most $e^{-\lambda /4}$, which can be made $O(n^{-2})$-small by picking a sufficiently-large constant *c* in $\lambda \ge c \log n$. Thus, we focus on the distribution of OPT/ALT islands in the final iteration of the phase, given that all islands have been in a non-LOST state for at least $4c'n$ iterations, where $c' > 0$ is a positive constant chosen such that Lemma 8 can be applied after $c'n$ iterations.

Focusing on the final iteration, let *T* be a random variable denoting the number of iterations that have elapsed since the last migration which occurred. As migration occurs independently at random in each iteration with probability $p_\mathrm {mig}$, *T* is geometrically distributed, and also describes the number of iterations between any two subsequent migrations. When $T \ge c'n$, we can apply Lemma 8, and call the island model *sufficiently-mixed*: because no migration has occurred for a while, all non-LOST islands have at least a positive constant probability of being in the ALT and OPT states, independent of each other.

From the properties of the geometric distribution, we know the phase ends on a sufficiently-mixed iteration with probability at least $p_s \ge (1 - n^{-1.5})^{c' n} \ge 1 - c'/\sqrt{n}$ (using Bernoulli’s inequality), and that either the phase transition or at least one of the last three migrations occurred on a sufficiently-mixed iteration with probability at least $1 - (1 - p_s)^4 = 1 - O(n^{-2})$. Thus, across all *n* phase transitions, we can conclude that with probability $(1 - O(n^{-2}))^n \ge 1 - O(1/n)$, there is a sufficiently-mixed iteration among the last $3c'n$ iterations of each phase, and either the phase transition, or one of the preceding three migrations occurs on a sufficiently-mixed iteration.

We now distinguish between two cases, depending on whether the phase transition occurred on a sufficiently-mixed iteration. If this is the case, as it is for the majority of the *n* phases, we will argue that this directly implies that at least one island will have OPT as its best-so-far individual and will keep tracking the oscillating optimum through the phase transition. If the phase transition does not occur on a sufficiently-mixed iteration, at it does for a $O(n^{0.5})$-minority of the phases, we will show that, with high probability, at least one of the three migrations preceding the phase transition occurred on a sufficiently-mixed iteration, and there will exist a segment of at least 4 islands with OPT as their best-so-far solution, and that at least one of these islands remains in the OPT state until the phase transition.

If the phase transition occurs on a sufficiently-mixed iteration, each island is in the OPT state with at least constant probability $p_O > 0$ per Lemma 8, and thus there exists a sufficiently large constant *c* in $\lambda \ge c \log n$ such that at least one island is in the OPT state when the phase transition occurs with probability $(1 - p_O)^\lambda = 1 - O(n^{-2})$.

If the phase transition does not occur on a sufficiently-mixed iteration, we look back to the last migration occurring on a sufficiently-mixed iteration. With probability $1-O(n^{-2})$, this migration occurs at most $3c'n$ iterations before the phase transition, and is followed by at most two other migrations. We divide the ring into $\lambda /4$ segments of 4 islands each, and focus on the probability $p_s$ that, in a given segment, all four islands are in the OPT state when the last sufficiently-mixed migration occurs, and no migration occurs on any of the four islands between the last sufficiently-mixed migration and the phase transition.

By Lemma 8, each island is in the OPT state independently with at least constant probability $p_O > 0$ during the sufficiently-mixed migration, and thus each segment consists entirely of OPT islands immediately before this migration with probability at least ${p_O}^4 = \varOmega (1)$. Additionally, no island in the segment is affected by mutation in the remaining $3c'n$ iterations with probability at least $(1-p_\mathrm {mut})^{3c'\,n} = \varOmega (1)$.

Thus, with constant probability $p_s > 0$, any given segment of 4 islands consists of only islands in the OPT state immediately prior to the last sufficiently-mixed migration, and is not affected by mutation until the phase transition. The fourth island in such a segment will remain in the OPT state until the phase transition: the closest island in the ALT state is at least four migrations away, while at most three migrations will occur prior to the phase transition, and migration will not occur on any island in the segment. Therefore, there exists a constant *c* for $\lambda \ge c \log n$ which ensures that with probability $(1 - p_s)^{\lambda /4} \ge 1 - n^{-0.25 c \log p_s} \ge 1 - n^{-2}$, at least one island will still track the oscillating optimum following the phase transition.

We can then combine the failure probabilities of the considered events across *n* phases: with probability $O(n^{-2})$, too few migrations occur to ensure that all islands are in a non-LOST state $4c'n$ iterations before the phase transition, with probability $O(n^{-2})$, there is no sufficiently-mixed iteration in the final $3c'n$ iterations before the phase transition, and with probability $O(n^{-2})$, none of the $c\log n$ islands are in the OPT state during the phase transition. Using a union bound, the simplified island model is able to track the optimum through *n* phases with probability at least $1 - O(n \cdot n^{-2}) = 1 - O(1/n)$. $\square$

We note the simplified island model is able to track the optimum even if the individual preferred by the next phase is not favored by the random oscillation, i.e. $0< p_\mathrm {OPT} < 1/2$. This also implies that with any constant $1/2< p_\mathrm {OPT} < 1$, at least one island will be in the ALT state during each of *n* phase transitions with high probability: thus, in this setting, the simplified island is able to guarantee some level of diversity among the island population.

It is possible to extend the proof of Theorem 12 to accommodate $p_\mathrm {mig} = n^{-(1+\epsilon )}$ for any positive constant $\epsilon > 0$. Such a change would increase the number of migrations that might occur between the last sufficiently-mixed migration and the phase transition to a larger constant. To accommodate this, the length of the OPT segments that need to exist immediately prior to the sufficiently-mixed migration would also need to be increased to a larger constant. This, in turn, may require the constant *c* in $\lambda = c \log n$ to be increased to maintain the same overall failure probability.

## Conclusion

We have demonstrated using rigorous analysis that there exist choices of parameters for the simplified island model for which a complete migration topology as well as all topologies with small logarithmic diameter with high probability result in a failure to track the oscillating optimum through all *n* phases. In the same settings, using a unidirectional ring migration topology of diameter $c\log n$, where $c>0$ is a sufficiently large constant, allows the optimum to be tracked through all *n* phases with high probability. This example illustrates that a less dense migration topology can mitigate the effects of migration occurring during unfavorable iterations of an oscillating fitness function, reducing the need to rely on problem-specific knowledge as in [17]. Moreover, the analysis reveals a crucial dependency of the efficiency of the model on the topology’s diameter, for which we have established a sharp-threshold result. At the other extreme, we have also proven that denser migration topologies may be advantageous if migration occurs only rarely, as in this setting the ring topology may not allow lost islands to be recovered quickly enough to replenish those which lose track of the oscillating optimum during phase transitions.

While this paper introduced and derived results based on the simplified island model, we believe that the presented results could be transferred to the original setting of $(1+1)$ EA islands tracking the original Maze function.

In future work, it would be useful to provide a more precise bound on the graph diameter threshold where the simplified island model transitions to being able to track the optimum through all *n* phases. Additionally, the presented results could be extended to less extreme settings of $p_\mathrm {mig}$, building on the initial result of “moderately frequent migration” considered in Sect. 5, which states that any constant $p_\mathrm {OPT} > 0$ is sufficient when $p_\mathrm {mig} = n^{-1.5}$ and the number of islands is at least logarithmic.

We note that while our theoretical analysis does not prove this directly, our experiments from Sect. 3.3 suggest that $p_\mathrm {mig} = 1$ combined with a low value of the product $\lambda \cdot p_\mathrm {mut}$ actually leads to a reduction in population diversity, with the majority of the islands settling on OPT as their current-best solution, rather than achieving a $p_\mathrm {OPT}$-like balance between OPT and ALT islands. We conjecture that such a balance could be achieved when using moderate migration probabilities.
